# Performance of Epworth Sleepiness Scale and tiredness symptom used with simplified diagnostic tests for the identification of sleep apnea

**DOI:** 10.5935/1984-0063.20190095

**Published:** 2019

**Authors:** Eduardo Borsini, Magalí Blanco, Silvana Schonfeld, Glenda Ernst, Alejandro Salvado

**Affiliations:** 1 Hospital Británico de Buenos Aires, Sleep an Ventilation Unit - Buenos Aires - Buenos Aires - Argentina.; 2 Hospital Británico de Buenos Aires, Respiratory Medicine Center - Buenos Aires - Buenos Aires - Argentina.; 3 Hospital Británico de Buenos Aires, Teaching and Research Department - Buenos Aires - Buenos Aires - Argentina.

**Keywords:** Sleep Apnea Syndromes, Hypersomnia, Sleepiness, Surveys and Questionnaires

## Abstract

**Objective:**

To compare the performance of Epworth Sleepiness Scale (ESS) and tiredness symptom with the apnea-hypopnea index (AHI) in a population referred to home sleep tests.

**Materials and Methods:**

This correlational study assessed adult patients through questionnaires and respiratory polygraphy (RP). We estimated sensitivity (S), specificity (Sp), predictive values (PV), odds ratio (OR) adjusted multivariate model and area under ROC curve for each sex and severity.

**Results:**

We analyzed 4424 patients, 2761 (62.4%) men and 1663 women, aged 53.6 (42-65 years old) with BMI of 31.3 (25.5-36.1). 78.4% had AHI >5 events/hour. RP (ev/h) indicators were (men vs. women): AHI of 22.8±19.2 vs. 13.2±13.3, ODI of 22.7±19.9 vs. 14.0±13.7, and T <90%: 19.3±26.1 vs. 15.6±25.3. Men presented higher severity levels, night-time hypoxemia and CPAP indications (52.2 vs. 29.2%) *p*<0.0001. ESS > 10 was found in 25% of population: 8±5.15 in men vs. 7.6±5.1 in women, *p*<0.001. 12% of men (as compared to 31.5% of women) with ESS > 10 had a normal AHI. 72% of women reported tiredness (vs. 66.1% of men). The R2 between Epworth Scale and AHI showed: 0.022 (CI95%: 0.111-0.185) *p*<0.0001 in men and 0.0019 (CI95%: -0.004 to 0.092) *p*>0.074 in women. Logistic regression showed Epworth Sleepiness Scale >10 for each AHI severity category (OR between 1.38 and 1.31 with *p*<0.05) and tiredness for AHI >30 ev/h only in men (*p*<0.004).

**Conclusions:**

Epworth Sleepiness Scale >10 demonstrated a low screening performance only when present in male patients. Tiredness performed worse. Due to its limited value in the identification of sleep apnea patients, subjective somnolence should be considered in the context of an objective evaluation.

## INTRODUCTION

Obstructive sleep apnea-hypopnea syndrome (OSA) has emerged as a public health concern due to its high prevalence in the general population and the associated high morbidity and mortality rates^1^.

OSA diagnostic criteria are based on an Apnea-Hypopnea Index (AHI) of >5 events per hour (ev/h) associated with excessive daytime sleepiness and/or cardiovascular or metabolic comorbidities. In middle-aged individuals, OSA prevalence^2^^,^^3^ was estimated at 5-9%. Nevertheless, in according to recent data^4^, OSA overall population prevalence ranges from 9% to 38%, lying close to 28% in Latin America^5^. Such finding calls for pragmatic diagnostic strategies^6^.

Traditionally, OSA diagnosis is confirmed with polysomnography (PSG) though a duly validated respiratory polygraphy (RP) is also accepted in populations with a high/low clinical likelihood of suffering from OSA^7^^,^^8^.

Excessive daytime sleepiness is a relevant symptom because of its direct impact on patients’ quality of life^9^ and traffic accidents^10^^-^12, in addition to be a significant marker of poor sleep quality^13^^,^^14^. However, not all patients with sleep disorders report excessive daytime sleepiness and individuals with high PSG AHI values often do not report this symptom at all^15^. On the other hand, excessive daytime sleepiness can also result from inappropriate sleep habits or psychiatric disorders, like depression^16^. In fact, lifestyle-related chronic sleep deprivation is one of the most frequent causes of daytime sleepiness^17^.

Current tools to assess excessive daytime sleepiness include self-administered subjective scales based on validated questionnaires against reference methods and objective tests like the Multiple Sleep Latency Test (MSLT) and the Maintenance of Wakefulness Test. But, besides limited availability in some contexts, they are time consuming and must be conducted in a proper setting.

In routine clinical practice, referral centers continue to use subjective sleepiness scales (ESS, Stanford Sleepiness Scale, and Pediatric Daytime Sleepiness Scale) because their implementation is inexpensive and does not require complex training/equipment.

The subjective sleepiness scale known as Epworth Sleepiness Scale (ESS)^18^ was first described by Murray W. Johns at Melbourne’s Epworth Hospital in Victoria, Australia, in 1991. Soon after its publication, it was translated into Spanish and its use became frequent worldwide among populations suffering from OSA^19^. Several studies have assessed the advantages and disadvantages of ESS with significantly different results.

A systematic literature search by Sil and Barr found 5 studies that reported a significant correlation between ESS and AHI and 11 studies that showed no correlation. In addition to the fact that the statistical tools used in these studies were heterogenous (Spearman’s rank correlation coefficient, logistic regression, multivariate analysis, ROC analysis, etc.), OSA was defined by either PSG AHI (with inconsistent cut-off points) or the comparison of ESS score with the multiple sleep latency test results^20^.

There is not much available information about ESS’ performance in OSA patients in Argentina. A validation study conducted in a small patient population using PSG^21^ reported that ESS scores >10 had a high positive predictive value (PPV). However, in our study, ESS showed modest performance in the identification of relevant OSA in 614 patients assessed through RP^22^.

In spite of these limitations, ESS scores >10-12 points are considered significant before a diagnostic test^8^^,^^23^^-^25. Finding a correlation between ESS scores and RP AHI in the population referred to our unit for sleep tests would allow us to detect sleepiness and thus, identify potential treatment candidates. The present analysis was implemented on a large patient sample referred to our center and assessed through home-based self-administered RP.

## OBJECTIVE

To compare the performance of ESS score, tiredness symptom and AHI in the population referred to home sleep tests.

## MATERIAL AND METHODS

### Population

This is a retrospective correlational study based on a convenience sampling conducted at a Respiratory Medicine Center between January 2013 and December 2018 (6 years) in adult patients suspected of sleep disorders on the grounds of three cardinal symptoms: frequent snoring, excessive daytime sleepiness, or partner-observed apneas.

All study procedures conducted on human participants were followed pursuant to the standards of the national and institutional research committee and the Declaration of Helsinki of 1964, as amended. The protocol was approved by the ethics and institutional review committee (protocol number: CRI#968).

**Figure 1:**
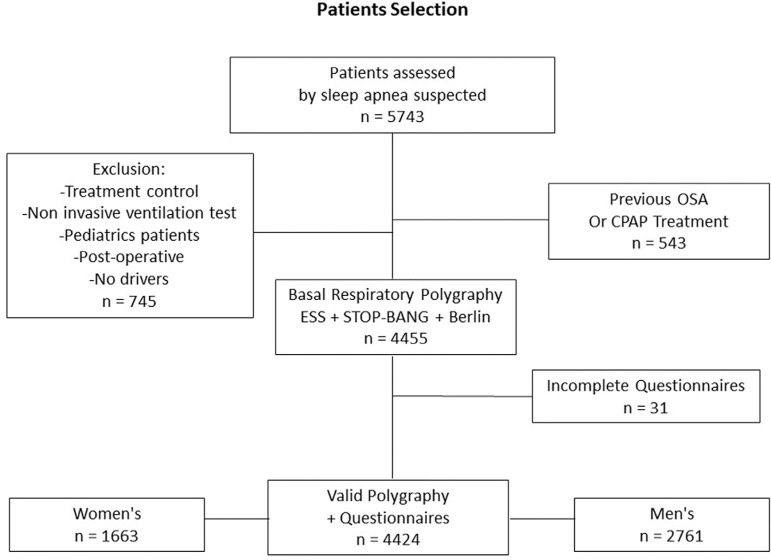
Patient`s selection flowchart.

Patients with more than one RP recording were included only once (Figure 1). Exclusion criteria applied to patients with daytime respiratory failure, heart failure, and those on mechanical ventilation or receiving supplemental oxygen. Records obtained during hospital stays or in post-surgical settings were also excluded.

### Respiratory Polygraphy

RP recordings were taken at night (1 night only) at patients’ homes using a self-administered technique (i.e. the patient sets and starts the RP device before falling asleep). Patients received proper training on the use of the device at the hospital the morning before the test. The training session lasted 20 minutes and was delivered by nurses with experience in sleep medicine. Additionally, patients received an instruction manual with pictures and information on how to set the device.

RP devices used in this study were Apnea Link Plus-Air (ResMed; Australia) and Alice Night One (Philips-Respironics; USA). All polygraph data from at least three basic signals: pulse oximetry, thoracic effort band, and nasal pressure canula. Ancillary signals included body position, actigraphy, and snoring.

Tracings were included/excluded through manual edition under AAMS standards^8^. Recordings with more than 240 minutes of valid total recording time (TRT) (>4 hours) met criteria for analysis. Apnea was defined as a drop of >80% in air flow and hypopnea as a reduction of >50% in air flow associated with a >3% drop in oxygen saturation for more than 10 seconds in both cases. AHI was calculated as the number of respiratory events (apneas and hypopneas) per hour. All data were estimated on the basis of total recording time valid for analysis after manual edition by expert pulmonologists. AHI >5 ev/h was considered clinically significant. AHI severity categories used were mild (6-14.9 ev/h), moderate (15-29.9 ev/h), and severe (≥30 ev/h).

### Questionnaires

When patients picked up the RP devices at our center, age, sex and anthropometric variables (body mass index [BMI] in kg/m^2^) were systematically collected. Obesity was defined as BMI >30 kg/m^2^. Daytime sleepiness was assessed with a validated Spanish translation of the current ESS version and OSA probability with Berlin and STOP-BANG (SBQ) questionnaires^15^^,^^26^^,^^27^. *Tiredness* (T) was assessed with the SBQ question specifically related to this symptom.

Each patient completed a printed copy of ESS before receiving the RP device^19^ and had to choose one option for each item (feeling sleepy or falling asleep in specific situations). Each item is scored from 0 to 3. The final score goes from 0 (no probability of falling asleep in any described situation) to 24 (high probability of falling asleep in all 8 situations described).

Non-drivers or visually impaired patients who were not able to complete ESS were not included.

### Statistical Analysis

A frequency histogram and a Kolmogorov-Smirnov test were used to assess the distribution of study variables. Quantitative variables were expressed as standard deviation and mean values and qualitative variables as absolute values and percentages.

Odds Ratio (OR) was used to calculate Sensitivity (S), Specificity (Sp), Positive Predictive Value (PPV) and Negative Predictive Value (NPV).

To include variables correlating ESS scores >10< and T (*tiredness*) for the identification of patients through AHI severity in a logistic regression model, we conducted a multivariate analysis and a Student’s t test or χ^2^ test/Fisher Test for quantitative or qualitative variables respectively. Once prediction variables were obtained, we used a multivariate forward stepwise analysis. The dependent variables were: *Epworth Sleepiness Scale Value* (dichotomous) and *tiredness* (present or absent); the independent variables being: sex, BMI (> or <30 kg/m^2^), history of hypertension, depression diagnosis, sex, age and Berlin questionnaire (high or low risk) to obtain odds ratio (OR) and confidence interval (CI 95%) for AHI severity classification. Finally, ROC (AUC-ROC) curves were estimated using the Hosmer-Lemeshow goodness-of-fit test. A *p* value <0.05 was considered statistically significant.

The commercial software package SPSS 9.0 was used (SPSS Inc. Chicago. Illinois, USA) and Prism 7.04 (GraphPad, La Jolla, CA).

## RESULTS

The study included 5743 patients. After the selection process (Figure 1), we analyzed data from a total of 4424 patients, 2761 (62.4%) men and 1663 women. Median age was 53.6 years old (42-65) and BMI was 31.3 kg/m^2^ (25.5-36.1). The most frequently reported symptoms were snoring in men and sleepiness in women. Table 1 summarizes population characteristics per sex and frequent symptoms according to SBQ.

**Table 1:** Characteristics of study population and frequent symptoms.

	All	Women	Men	p*
Number of patients (n; %)	4424	1663 (37.6)	2761 (62.4)	
Age (years)	53.6 (42-65)	53.4 (43-65)	54 (43-65)	0.239
Age >50 years old (n; %)	2702 (61.1)	1018 (61.2)	1684 (61)	0.883
Body Mass Index (BMI; kg/m^2^)	31.3 (25.5)	31.1 (26.6-)	30.0 (27.1-)	0.001
BMI ≥30 kg/m2	1424 (32.2)	621 (37.3)	803 (29.1)	< 0.001
Neck circumference >40 cm (M) or 43 cm	2571 (58.1)	684 (41.1)	1887 (68.3)	< 0.0001
Hypertension	2297 (51.9)	789 (47.4)	1508 (54.6)	< 0.0001
High Risk Berlin Questionnaire	2541 (57.4)	953 (57.9)	1588 (57.8)	0.004
Prevalence of AHI >5 ev/h (n; %)	3469 (78.4)	1131 (69.2)	2338 (84.6)	< 0.001
ESS	7.9 ± 1.9	7.6 ± 5.1	8 ± 5.15	0.001
Frequent snoring	2829 (63.9)	973 (58.5)	1856 (67.2)	< 0.0001
Frequent sleepiness	3023 (68.3)	1198 (72)	1825 (66.1)	< 0.0001
Observed apneas	1998 (45.2)	570 (34.3)	1428 (51.7)	< 0.0001

Data presented as median values and percentiles 25-75 % or as mean and standard deviation (±). Data presented as n (%).

* Pearson's chi-squared test. BMI: body mass index in kg/m^2^; ESS: Epworth Sleepiness Scale; AHI: apnea-hypopnea index per hour of recording; ev/h: events per hour.

**Figure 2:**
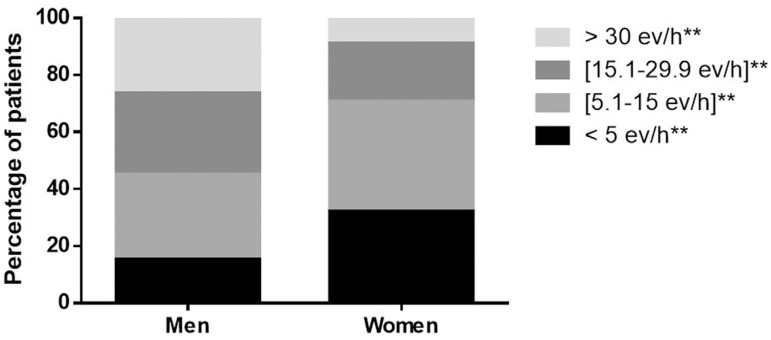
Distribution of severity based on Respiratory Polygraphy AHI (* represents statistical significance for each decimal digit).

AHI >5 ev/h was found in 78.4% of patients and severe OSA was observed in 26.4% in men and 9% in women (*p*<0.001). Conventional RP indicators (ev/h) showed (men *vs.* women): AHI: 22.8±19.2 *vs.* 13.2±13.3; ODI: 22.7±19.9 *vs.* 14.0±13.7; and T <90% (as % of TRT): 19.3±26.1 *vs.* 15.6±25.3 (Figure 2).

Male patients presented higher levels of severity, nighttime hypoxemia, and higher rates of CPAP therapy prescription (52.2 *vs.* 29.2%) *p*<0.0001.

**Figure 3:**
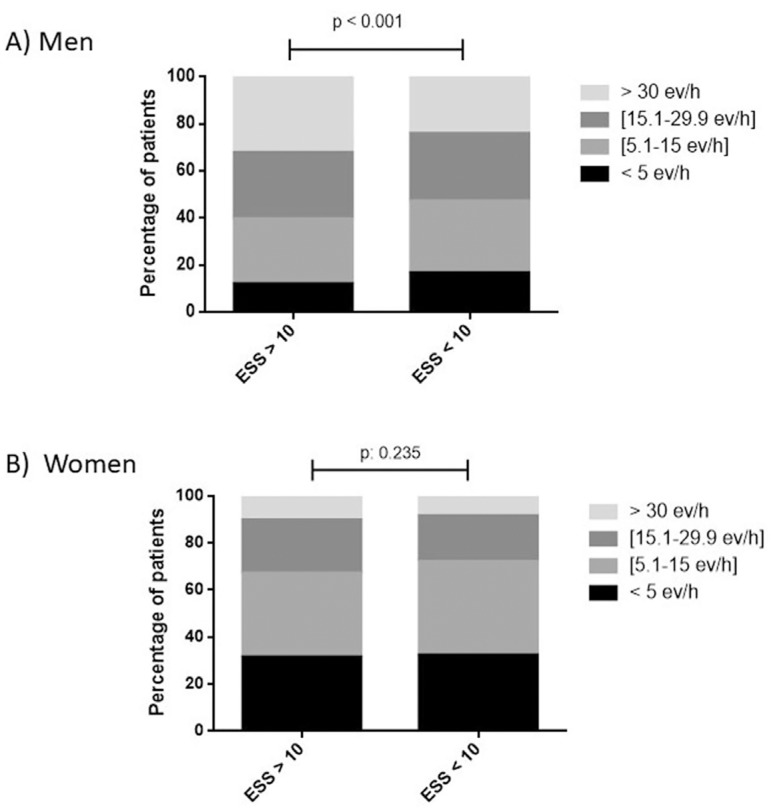
Distribution of ESS scores >10 with regard to Respiratory Polygraphy AHI grouped by severity (A: Men and B: Women).

We found ESS scores >10 in 25% of the population and a statistical difference between men and women of 8±5.15 *vs.* 7.6±5.1, which increased with AHI severity in men (Figure 3).

12% of men *vs.* 31.5% of women with ESS scores >10 had a normal AHI. *Tiredness* was reported by 72% of women and 66.1% of men. Inconsistency between (T: *Tiredness*) and ESS scores >10 was present in 50% and 40.7% of women and men respectively (Tables 2 and 3).

**Table 2:** Distribution of the symptom "Tiredness" for each sex with regard to respiratory polygraphy result.

Sex	Tiredness	AHI				Total (n)	n	*p
		< 5 ev/h	5.1-15 ev/h	15.1-29.9 ev/h	> 30 ev/h			
Men	+	256 (14)	537 (29.4)	507 (27.8)	525 (28.8)	1825 (66.1)	2761	0.001
	-	167 (17.8)	285 (30.4)	279 (29.8)	205 (22%)	936 ((33.9)		
Women	+	372 (31)	471 (39.3)	245 (20.5)	110 (9.2)	1198 (72)	1663	0.607
	-	160 (34.4)	471 (39.3)	93 (20)	39 (8.4)	465 (30)		

* Pearson's test for multiple categories per sex; ESS: Epworth Sleepiness Scale; AHI: apnea-hypopnea index per hour of recording.

**Table 3:** Inconsistency between subjective daytime sleepiness (ESS >10) and tiredness for each sex.

	Epworth Subjective Sleepiness Scale			
	< 10		> 10	
Sleepiness	Men	Women	Men	Women
**+**	1125 (40.7)	781 (50)	700 (25.3)	417 (25)
**-**	813 (29.4)	425 (25.5)	123 (4.5)	40 (2.4)

Correlation between ESS and AHI showed a R^2^ of 0.022 (CI95%: 0.111-0.185) *p*<0.0001 in men and 0.0019 (CI95%: -0.004 to 0.092) *p*>0.074 in women (Figure 4).

**Figure 4:** Correlation between ESS score and AHI (ev/h) from home sleep test. (A: Men and B: Women). Find statistical significance below

Table 4 presents S, Sp and AUC-ROC for each severity category based on AHI, showing that ESS scores >10 and Tiredness have a modest performance and a limited screening value in men.

**Table 4:** Sensitivity, Specificity, and area under the ROC curve (UAC-ROC) for severity categories based on AHÍ with regard to symptoms of sleepiness (ESS >10) and tiredness (T: obtained from SBQ) for each sex.

Variables		CATEGORIES OF SEVERITY BASED ON AHI																	
		5.1-15 ev/h						15.1-29.9 ev/h						> 30 ev/h					
		S	Sp	PPV	NPV	ROC	p	S	Sp	PPV	NPV	ROC	p	S	Sp	PPV	NPV	ROC	p
Men	ESS >10	88.0	16.7	85.7 (84.1-87.3)	10 (15.5-25.1)	0.52	0.009	60.3	47.4	86.7 84.8-88.5	17.4 15.1-20.1	0.53	0.002	32.1	76.1	87 81.2-90.5	13 10.1-19.1	0.54	0.0001
	Tiredness	85.9	17.8	85.9 (83.9-87.8)	16.1 (13.9-18.6)	0.52	0.03	56	47	87.8 83.2-85.5	14 15.1-20.9	0.54	0.001	28.6	77.9	88.2 (85.4-90.6)	16.2 (14.5-18.1)	0.54	0.002
Women	ESS >10	68.5	32.2	70 (67.2-72.7)	30 (26.1-34.1)	0.50	0.79	32.8	72.1	73.3 (69.3-77.1)	28.7 (28.8-34.3)	0.50	0.06	10.1	91.5	72.2 (64.6-78.9)	30.2 (27.9-32.6)	0.50	0.34
	Tiredness	69	34.4	70.1 (68.2-73.6)	31 (27.1-33.9)	0.51	0.19	29.6	71.6	75 (70.9-78.1)	25.1 (28.0-34.1)	0.51	0.61	9.2	91.6	70 (62.0-77.2)	30 (27.7-32.4)	0.51	0.60

ESS: Epworth Sleepiness Scale; AHI: apnea-hypopnea index per hour of recording; Ev/h: events per hour. S: Sensitivity; Sp: specificity; PPV: positive predictive value; NPV: negative predictive value; ROC: area under the curve. (*Confidence Interval between parenthesis*: 95%).

Adjusted Logistic Regression showed the predictive capacity of ESS scores >10 for each severity category (OR between 1.38 and 1.31) and sleepiness for AHI >30 ev/h exclusively in men (Table 5).

**Table 5:** Adjusted Logistic Regression for categories of severity based on AHI with regard to symptoms of sleepiness and tiredness for each sex.

Variables		CATEGORIES OF SEVERITY BASED ON AHI					
		5.1-15 ev/h		15.1-29.9 ev/h		>30 ev/h	
		**OR (IC 95%)**	**p**	**OR (CI 95%)**	**p**	**OR (CI 95%)**	**p**
Men	ESS >10	1.38 (1.07-1.77)	0.010	1.31 (1.11-1.56)	0.002	1.38 (1.15-1.67)	0.001
	Tiredness	1.23 (0.98-1.53)	0.06	1.13 (0.96-1.33)	0.131	1.32 (1.09-1.60)	0.004
Women	ESS >10	0.98 (0.77-1.25)	0.927	1.26 (0.99-1.60)	0.06	1.18 (0.80-1.72)	0.389
	Tiredness	1.16 (1.57-2.31)	0.196	0.99 (0.78-1.27)	0.989	1.05 (0.70-1.56)	0.790

ESS: Epworth Sleepiness Scale; AHI: apnea-hypopnea index per hour of recording. Ev/h: events per hour; OR: Odds Ratio. (*Confidence Interval between parentheses*: 95%).

## DISCUSSION

This study carried out in a large sample shows a low correlation between subjective somnolence symptoms and objective indicators obtained by the home sleep test, which show the limited usefulness of subjective questions to identified sleep apnea patients or estimate their severity.

Several studies have assessed the relationship between ESS score and AHI in different populations^28^^-^32 and cultures, which makes it difficult to compare or extrapolate results^20^. The use of different analytical methods in heterogeneous populations distracts our attention from a key question: Are the subjective symptoms of sleepiness good predictors of sleep apnea?

Patients suffering from sleep apnea may ignore the alterations in their breathing patterns during sleep, as clinical signs are often reported by their roommates or partners. Therefore, this population is hardly aware of the cardiovascular and/or metabolic risk they face.

This study was conducted on patients suspected of OSA referred to our unit to undergo a specific sleep test. One fourth of our sample revealed ESS scores >10 and high OSA prevalence (>78%). It is worth considering that both the traditional classification of severity is only an approximation^33^ and the use of diagnostic RP may result in the underestimation of indicators^8^^,^^27^.

ESS questionnaire is routinely used to assess subjective sleepiness in patients with a clinical suspicion of OSA, though several scientific studies have consistently shown its low sensitivity^20^. In general, validations have been based on monitored PSG, rather than the now extensively used home-based RP^15^^,^^20^^,^^34^^,^^35^.

ESS is frequently used in our units and, therefore, we want to learn more about its performance in our population and its relationship with home-based RP results, another routine practice at our center.

While the American Academy of Sleep Medicine^8^ regards ESS scores >10 as ‘significant daytime sleepiness’ and the Spanish consensus document^7^ (2005) suggests a cut-off point of >12, the last version (2019) of the Argentine Consensus on Sleep-related Respiratory Disorders^36^ does not state ESS reference values for practice purposes.

In 2008, Rosenthal & Dolan^29^ assessed Epworth S and Sp in OSA diagnosis using PSG in a 268-patient population. They reported S of 66% for a cut-off point of 10 with an area under the curve of 0.60. Thus, they demonstrated ESS was inappropriate for OSA diagnosis, which is consistent with our results. Though, our sex-differential analysis reveals ESS performs better in men.

In a local study using PSG and a clinical questionnaire adjusted for age, BMI, and respiratory distress index, Nigro et al.^37^ stated women are less likely to report snoring and apnea. Likewise, they reported female sex was an independent predictor of sleepiness, though ESS >10 was not a predictor of OSA in line with our observations.

OR analysis for different ESS cut-off points shows that male patients with ESS scores >10 are at a higher risk of high AHI. Logistic regression adjusted for confounders like obesity, neck circumference, sex, depression, Berlin questionnaire risk, hypertension, and age showed an OR between 1.3 and 1.4 in men, with areas under the curve with limited discrimination value (0.63 to 0.64). Even though an OR of 1.4 is statistically significant, its clinical impact may be limited in the actual identification of patients with sleep apnea^20^.

Very few studies have used logistic regression and they have also found a low degree of correlation between ESS and AHI^25^^,^^34^. A British study that assessed a total of 238 patients with PSG^20^ obtained similar ROC curves with an area of 0.6, concluding that the ESS usefulness is limited.

Osman at al.^35^ state that ESS is not a good predictor of OSA based on its poor correlation with AHI and suggest daytime sleepiness can also exist in non-OSA snoring patients, which is consistent with our findings. However, it is worth noting that ESS scores >10 are associated with a higher probability of high AHIs in moderate to severe cases (candidates to receive CPAP therapy) with acceptable S (>75% in men and 91.5% in women) and high PPV (>70) in a context where this disorder is highly prevalent. These data are also consistent with those obtained by PSG^34^^,^^35^.

As is appreciated in the literature, the clinical correlation is of great value when simplified tests are used.

The use of daytime sleepiness and tiredness symptoms is widespread in different sleep units around the world, because it allows the clinician to check the correlation of sleep study results with self-reported symptoms. However, due to its low correlation with objective indicators, the usefulness of this strategy may only be verified in symptomatic patients.

### Study Limitations

Our study was conducted on patients from one single center using retrospective analysis, with the limitations inherent to this type of study design. Geographic, social, and cultural factors make it difficult to extrapolate our results to other populations and health systems.

The identification of respiratory events in RP recordings could also be a limitation of our study because AHI was calculated by manual reading. Thus, such index is the result of the addition of the number of apneas/hypopneas per hour of recorded time, i.e. a quotient of the number of observed events and time of exposure.

## CONCLUSIONS

ESS had a limited value in the identification of patients’ severity and should be considered in the context of an objective evaluation together with AHI. In our experience, ESS scores > 10 have a limited discrimination capacity and are useful especially in male patients. Tiredness as a symptom performed even worse and was useful only when reported by male patients with severe OSA. 
